# Largely Suppressed Magneto-Thermal Conductivity and Enhanced Magneto-Thermoelectric Properties in PtSn_4_

**DOI:** 10.34133/2020/4643507

**Published:** 2020-04-07

**Authors:** Chenguang Fu, Satya N. Guin, Thomas Scaffidi, Yan Sun, Rana Saha, Sarah J. Watzman, Abhay K. Srivastava, Guowei Li, Walter Schnelle, Stuart S. P. Parkin, Claudia Felser, Johannes Gooth

**Affiliations:** ^1^Max Planck Institute for Chemical Physics of Solids, 01187 Dresden, Germany; ^2^Department of Physics, University of California, Berkeley, CA 94720, USA; ^3^Department of Physics, University of Toronto, Toronto, Ontario M5S 1A7, Canada; ^4^Max Planck Institute of Microstructure Physics, 06120 Halle, Germany; ^5^Department of Mechanical and Materials Engineering, University of Cincinnati, Cincinnati, OH 45219, USA; ^6^Institute of Physics, Martin Luther University Halle-Wittenberg, 06120 Halle, Germany

## Abstract

Highly conductive topological semimetals with exotic electronic structures offer fertile ground for the investigation of the electrical and thermal transport behavior of quasiparticles. Here, we find that the layer-structured Dirac semimetal PtSn_4_ exhibits a largely suppressed thermal conductivity under a magnetic field. At low temperatures, a dramatic decrease in the thermal conductivity of PtSn_4_ by more than two orders of magnitude is obtained at 9 T. Moreover, PtSn_4_ shows both strong longitudinal and transverse thermoelectric responses under a magnetic field. Large power factor and Nernst power factor of approximately 80–100 *μ*W·cm^−1^·K^−2^ are obtained around 15 K in various magnetic fields. As a result, the thermoelectric figure of merit *zT* is strongly enhanced by more than 30 times, compared to that without a magnetic field. This work provides a paradigm for the decoupling of the electron and hole transport behavior of highly conductive topological semimetals and is helpful for developing topological semimetals for thermoelectric energy conversion.

## 1. Introduction

In recent years, thermoelectric effects have attracted considerable attention in the fields of materials science and solid-state physics and chemistry. Practically, solid-state thermoelectric conversion offers a promising solution for energy harvesting and cooling [1]. Furthermore, investigations of thermoelectric phenomena are important for understanding the fundamental transport behavior of quasiparticles in solid materials [2]. The thermoelectric efficiency of a material is gauged by the figure of merit, *zT* = *S*^2^*T*/*ρκ*, where *S*, *T*, *ρ*, and *κ* are the thermopower, absolute temperature, electrical resistivity, and thermal conductivity, respectively. The term *S*^2^/*ρ* is known as the thermoelectric power factor. Although the expression is simple, obtaining a high *zT* is a challenging task because these transport parameters are interrelated. As a compromise, heavily doped semiconductors with moderate electrical conductivity and thermopower values have become the most widely investigated thermoelectric systems [3–5]. Metals or semimetals, owing to strong coupling of *S*, *ρ*, and the electronic thermal conductivity *κ*_*e*_, have attracted much less attention for thermoelectric studies. The key to developing metals or semimetals for thermoelectric application lies in the decoupling of the three electrical transport parameters.

In a two-carrier semimetal system, the thermopower *S* is expressed as (*S*_*e*_*σ*_*e*_ + *S*_*h*_*σ*_*h*_)/(*σ*_*e*_ + *σ*_*h*_), where *e* and *h* indicate electrons and holes, respectively, and *σ* is the electrical conductivity. *S*_*e*_ and *S*_*h*_ generally have opposite signs and thus balance each other's contribution to *S*. Assuming the conduction and valence bands have symmetric structure and the Fermi level lies between them, the terms *S*_*e*_*σ*_*e*_ and *S*_*h*_*σ*_*h*_ will have similar values but opposite signs, so *S* will be almost zero. Therefore, semimetals with symmetric conduction and valence bands have generally been viewed as poor thermoelectric materials. One way to overcome this disadvantage is to look for semimetals with a large asymmetry between the conduction and valence bands, where the large disparity in their electron and hole effective masses could ensure a sizable *S* [6, 7]. Another recently proposed method of generating a large thermopower in topological Dirac/Weyl semimetals is to apply a sufficiently large magnetic field, under which the thermopower will grow linearly without saturation [8]. These proposals suggest feasible ways to tune the thermoelectric properties of topological semimetals.

When a conductive solid is placed under a longitudinal temperature gradient and a transverse magnetic field, two types of thermoelectric responses occur, i.e., the magneto-Seebeck effect in the longitudinal direction and the Nernst effect in the other transverse direction [9]. Both longitudinal and transverse thermoelectric effects have recently received attention in the studies of topological semimetals, specifically, studies of giant magnetic-field enhancement of the thermoelectric properties [10, 11] or exploration of the Berry curvature-related anomalous thermoelectric transport phenomena [12–18]. For instance, in the Dirac semimetal Cd_3_As_2_ [19], which has ultrahigh carrier mobility [20], anomalous magneto-Seebeck and Nernst effects were recently observed [12, 14], and the relationship between these anomalous transport properties with Berry curvature was discussed. Moreover, the magnetic-field-induced giant enhancement in both longitudinal and transverse *zT* values were found in Cd_3_As_2_ single crystals near room temperature [11, 21, 22]. NbP, one of the first type-I Weyl semimetals to be discovered [23–25], has recently attracted attention as a material platform for exploring the Weyl physics-related thermoelectric transport properties. Large magneto-thermopower and Nernst thermopower were observed in both single crystals and polycrystalline samples [10, 16, 26]. These findings demonstrate that topological semimetals provide fertile ground for exploring magnetic-field-mediated thermoelectric properties. Moreover, recent high-throughput searches have identified thousands of topological semimetals in the Inorganic Crystal Structure Database [27–29]. Among them, those with complex crystal structure or heavy elements would display high potential for magneto-thermoelectric conversion, as they could have an intrinsically low phonon thermal conductivity.

**Figure 1:**
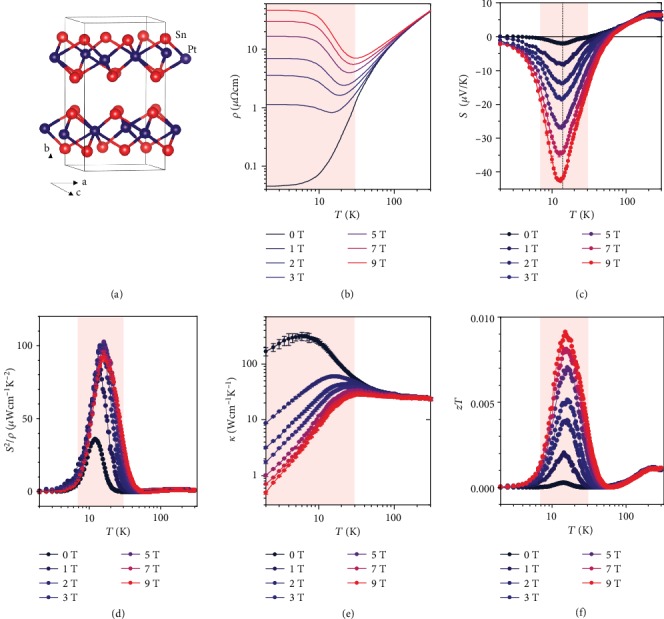
(a) Crystal structure of PtSn_4_. The chemical bonds indicate eight-fold Sn-coordinated Pt atoms. (b–f) Temperature dependence of electrical resistivity (b), thermopower (c), power factor (d), thermal conductivity (e), and *zT* (f) of PtSn_4_ single crystals under various magnetic fields.

In this work, we study the electrical and thermal transport properties of the Dirac semimetal PtSn_4_ under a magnetic field. The Dirac nodal arc structure and high magnetoresistance (MR) in PtSn_4_ suggest that the electrical properties will respond strongly to a magnetic field [30, 31]; furthermore, the layered structure with *a*–*c* planes stacked vertically along the *b* axis (Figure 1(a)) and low Debye temperature of 210 K [31] suggest a low phonon thermal conductivity, which however has not been reported. In our experiments, we used PtSn_4_ single crystals grown from a Sn-rich binary melt [31]. The growth process and chemical and structural characterizations are described in the Methods section. The electrical resistivity *ρ*, thermopower *S*, Nernst thermopower *S*_*xy*_, Hall resistivity *ρ*_H_, and thermal conductivity *κ* were measured at various magnetic fields, *B*, up to 9 T. The details of the measurement procedure are also presented in the Methods section. All the transport experiments were conducted within the *a*–*c* plane of the crystals, and the magnetic field is applied along the *b* axis. The transport properties exhibited a dramatic response to the magnetic field below 30 K. When the magnetic field was applied, a large power factor of 80–100 *μ*W·cm^−1^·K^−2^ and a very large suppression of the thermal conductivity appeared, which together contribute to an obvious increase in the peak *zT* at 15 K. Moreover, PtSn_4_ also exhibits a strong Nernst signal and a large Nernst power factor of 90 *μ*W·cm^−1^·K^−2^ at 9 T and 10 K. These findings on PtSn_4_ provide a good paradigm for tuning highly conductive topological semimetals for simultaneous longitudinal and transverse thermoelectric energy conversion.

## 2. Results and Discussion

In the first set of transport experiments at zero magnetic field, we confirm that the PtSn_4_ crystals exhibit the previously observed low electrical resistivity *ρ*. In Figure 1(b), the measured *ρ* is shown as a function of *T*. In agreement with the literature [31], *ρ*(*T*) at zero field increases linearly with *T* between 25 and 300 K, as expected for such semimetals, in which electron-phonon scattering is thought to dominate. Below 8 K, the resistivity starts to saturate, with a residual resistivity of 45 n*Ω*·cm at 2 K. Furthermore, a high residual resistance ratio of *ρ*(300 K)/*ρ*(2 K) ≈ 1000 is observed, which, together with the nonsaturating MR up to 9 T, indicates a long carrier mean free path.

The absolute thermopower of PtSn_4_ at 0 T is much lower than that of typical thermoelectric semiconductors. *S*(*T*) is positive at high *T* and exhibits a *p*–*n* transition upon cooling (Figure 1(c)), indicating that PtSn_4_ is a two-carrier semimetal system. When the magnetic field is applied, *S* depends strongly on *B* at approximately 14 K, in agreement with the literature [31]. As a result, a moderate thermopower *S*≈−40 *μ*V·K^−1^ at 9 T is obtained. Even though the magnetic field also induces strong MR, large peak power factors of 80–100 *μ*W·cm^−1^·K^−2^, calculated by *S*^2^/*ρ*, are obtained under various magnetic fields (Figure 1(d)). Recalling that good thermoelectric materials generally have relatively low power factor, for example, Bi_2_Te_3_ (30–50 *μ*W·cm^−1^·K^−2^) [32], PbTe (≈20 *μ*W·cm^−1^·K^−2^) [33, 34], SnSe (≈10 *μ*W·cm^−1^·K^−2^) [35], CoSb_3_ (30–50 *μ*W·cm^−1^·K^−2^) [36], and half-Heusler compounds (30–50 *μ*W·cm^−1^·K^−2^) [37], this large *S*^2^/*ρ* observed in PtSn_4_ under a magnetic field indicates its potential for thermoelectric conversion.

A large enhancement in *S*^2^/*ρ* is not the only required thermoelectric property; besides, the thermal conductivity should also be suppressed. We further measured the thermal conductivity *κ* of our samples as a function of temperature under various magnetic fields (Figure 1(e)). The experiments were performed with open electrical contact to prevent electric current flow. *κ*(*T*) indicates a metallic *T* dependence, which is consistent with the *ρ* and *S* measurements. Upon warming from 2 K, *κ*(*T*) increases linearly with *T* owing to the dominant impurity scattering. Near 8 K, *κ*(*T*) reaches a maximum and then starts to decrease. Remarkably, *κ*(*T*) exhibits a rapid decay with increasing *B*. A decrease in *κ*(*T*) by more than 2 orders of magnitude is observed at 9 T and 2 K. This huge suppression of *κ*(*T*) is a result of the giant MR, which indicates that charge carriers, rather than phonons, dominate the thermal transport in PtSn_4_. Owning to the magnetic-field-induced giant reduction in *κ* and enhancement of *S*^2^/*ρ*, PtSn_4_ exhibits a peak *zT* of 0.009 at 9 T and 15 K (Figure 1(f)), which is more than 30 times higher than the *zT* at zero field. Note that this peak in *zT* is obtained at a very low temperature of 15 K. The *z* value, a more intrinsic material parameter reflecting the thermoelectric potential, is 6 × 10^−4^ K^−1^ for PtSn_4_ at 9 T and 15 K, which is close to that of good thermoelectric materials, for example, Bi_2_Te_3_ (≈40 × 10^−4^ K^−1^) [32], PbTe (≈25 × 10^−4^ K^−1^) [33, 34], CoSb_3_ (≈20 × 10^−4^ K^−1^) [36], and half-Heusler compounds (≈10 × 10^−4^ K^−1^) [37]. More importantly, this very large increase in the peak *zT* of PtSn_4_ results from the simultaneous improvement of *S*^2^/*ρ* and suppression of *κ* with increasing *B*, which provides a good paradigm for the decoupling of the electrical and thermal transport properties.

To understand the magnetic-field-induced changes in the electrical transport properties, we further investigated the Hall resistivity *ρ*_H_ as a function of *B* at various *T*. Upon cooling from 300 K, *ρ*_H_ has a positive slope as a function of *B*, indicating that holes dominate the transport. Below 50 K, the slope of *ρ*_H_(*B*) reverses its sign at high fields. Because *dρ*_H_(*B*)/*dB* changes sign at low temperatures, especially around 25 K, it is tempting to associate this phenomenon with the transport contributions of both electrons and holes, which is also reflected by its thermopower at similar temperatures with almost 0 *μ*V·K^−1^. Moreover, the Fermi surface of PtSn_4_ was calculated using density functional theory (Figure S1) and also indicates that PtSn_4_ is a two-carrier system with multiple electron and hole pockets.

**Figure 2:**
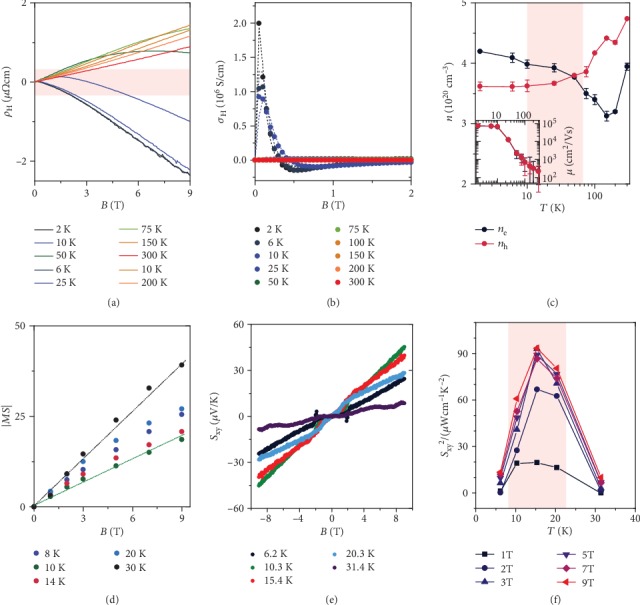
(a) Hall resistivity *ρ*_H_(*B*) at various *T*. (b) Hall conductivity *σ*_H_ = *ρ*_H_/(*ρ*_H_^2^ + *ρ*^2^) at various *T*. The symbols represent the experimental data, and the dotted lines are fits according to a two-carrier model. (c) Carrier densities *n*_*e*_, *n*_*h*_ and mobilities *μ*_*e*_, *μ*_*h*_ as a function of *T* for electrons and holes, respectively. The lines are guides to the eyes. The error bars represent the fitting error. (d) Absolute magneto-thermopower ∣MS∣ = ∣[*S*(*B*) − *S*(0 T)]/*S*(0 T)| at various *T*. The dotted lines indicate linear fits. (e) Nernst thermopower *S*_*xy*_ as a function of *B* at various temperatures. (f) Nernst power factor *S*_*xy*^2^_/*ρ* as a function of *T* at various *B*.

For a more quantitative analysis of the carrier densities and mobilities, we calculated the Hall conductivity, *σ*_H_ = *ρ*_H_/(*ρ*_H_^2^ + *ρ*^2^) (Figure 2(b)) using a two-carrier model:

(1) $$\begin{array}{c} \sigma_{\text{H}}\left(B\right)=\left[\frac{n_{h}^{2}\mu_{h}^{2}}{1+\mu_{h}^{2}B^{2}}-\frac{n_{e}^{2}\mu_{e}^{2}}{1+\mu_{e}^{2}B^{2}}\right]eB. \end{array}$$

This model enables us to obtain the temperature-dependent average carrier densities, *n*_*e*_, *n*_*h*_ and the carrier mobilities *μ*_*e*_, *μ*_*h*_ for the electron and hole pockets, respectively. The best fits to our data indeed reveal that our PtSn_4_ samples exhibit parallel transport of both electrons and holes across the full temperature range investigated (Figure 2(c)), with a hole excess above 50 K and an electron excess below. The electron and hole mobilities are very high (Figure 2(c), inset) and are comparable to those of the other topological semimetals [20, 25], in which the high mobility are responsible for the giant MR observed at low *T* (Figure 1(b)).

After determining the carrier densities, we further investigated the thermopower as a function of *B* around the temperatures at which *S* peaks. As shown in Figure 2(d), a dramatic enhancement of *S* occurs with increasing *B*. The absolute magneto-thermopower, ∣MS∣ = ∣[*S*(*B*) − *S*(0 T)]/*S*(0 T)∣, becomes significant at higher magnetic fields (*B* > 3 T), exhibiting an approximately linear relationship with *B*. The linear relationship at high magnetic fields can be understood in terms of the increase in the electron-phonon scattering rate with *B* [38], yielding |MS| ≈ *ћω*_c_/*k*_*B*_*T* for *ћω*_c_ > *k*_*B*_*T*, where *ћ* and *k*_*B*_ are the reduced Planck and Boltzmann constants, respectively. In addition, *ω*_c_ = *eB*/*m*_*e*/*h*_^∗^ denotes the cyclotron frequency, where *e* is the elementary charge. For an average effective mass, *m*_*e*/*h*_^∗^ = 0.2*m*_*e*_, a linear relation between |MS| and *B* is expected above 3 T, in agreement with the experiments.

Considering the large carrier mobilities below 30 K and the strong response of the longitudinal thermopower to a magnetic field, it is tempting to check for the transverse thermoelectric response, i.e., the Nernst effect. The Nernst thermopower *S*_*xy*_ of PtSn_4_ at various temperatures was further measured by a standard one-heater, two-thermometer configuration. As shown in Figure 2(e), *S*_*xy*_ displays a normal Nernst signal, namely, an approximately linear increase upon magnetic-field application, and no saturation is found at magnetic fields of up to 9 T. At 9 T and 10.3 K, *S*_*xy*_ reaches a moderate value of 45 *μ*V·K^−1^. Furthermore, because the electrical resistivity in the *a*–*c* plane is approximately isotropic [39], the Nernst power factor can be estimated as *S*_*xy*^2^_/*ρ* (Figure 2(f)). Like the conventional power factor (Figure 1(d)), the Nernst power factor also peaks at approximately 15 K. A maximum value of approximately 90 *μ*W·cm^−1^·K^−2^ is obtained at various magnetic fields, which is approximately 3 times larger than the value obtained in polycrystalline bulk NbP [10].

**Figure 3:**
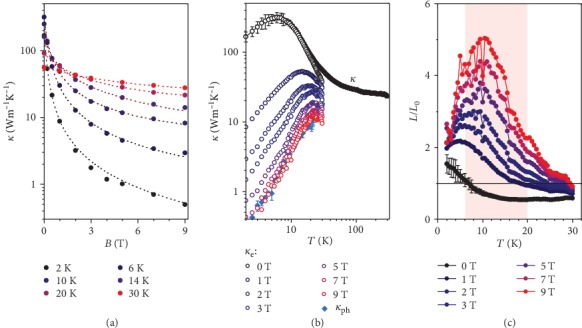
(a) Thermal conductivity *κ* as a function of *B* at various *T*. The dotted lines represent fits of *κ*(*B*, *T*) = *κ*_*ph*_(*T*) + *κ*_*e*_(*T*)/(1 + *ηB*^*s*^) to the data. (b) Extracted *κ*_*ph*_ and *κ*_*e*_(*B*) under various magnetic fields. (c) Lorenz number *L*, scaled by the Sommerfeld value *L*_0_, as a function of *T* at various *B*.

The thermal conductivity *κ* as a function of *B* is shown in Figure 3(a). At 2 K, as the magnetic field increases, *κ*(*B*) decreases by more than 2 orders of magnitude. The phonon thermal conductivity of a metallic system typically does not change significantly under a magnetic field. The very large decrease in *κ*(*B*) is thus a result of the large MR (Figure 1(b)), which then makes it possible to separate the phonon and electron components of the thermal conductivity. An empirical expression, *κ*(*B*, *T*) = *κ*_*ph*_(*T*) + *κ*_*e*_(*T*)/(1 + *ηB*^*s*^) [40, 41], was employed to extract the phonon thermal conductivity, *κ*_*ph*_(*T*), and electron thermal conductivity, *κ*_*e*_(*T*), at 0 T, where *η* and *s* are related to the thermal mobility and scattering mechanism, respectively. The obtained fitting parameters are shown in Table S1. Because this method is generally effective for large *μB*, only the *κ*(*B*, *T*) data below 30 K were fitted. The fitted lines match the experimental data well, as shown in Figure 3(a), indicating the feasibility of this method. The extracted *κ*_*e*_(*B*, *T*) decreases continuously with increasing *B*, as shown in Figure 3(b). At *B* = 0 T, *κ*_*e*_(*T*) is significantly larger than *κ*_*ph*_(*T*), demonstrating that PtSn_4_ is indeed a metallic system in which charge carriers dominate the electrical and thermal transport properties. The Lorenz number *L* is further calculated according to the Wiedemann–Franz (WF) law [1], as shown in Figure 3(c). At *B* = 0 T, this is a downward violation of the WF law, whereas an upward violation is found when the magnetic field is applied. Violations of the WF law typically indicate inelastic electron-phonon scattering or hydrodynamic transport, as recently found in the Weyl semimetal WP_2_ [42, 43]. Moreover, in a very recent work on another Weyl semimetal system, TaP, Han et al. also observed an upward violation of the WF law [40]. The reason is still not clear, but it seems that violation of the WF law under a magnetic field is common in topological semimetals.

## 3. Conclusions

In summary, measurements of the electrical and thermal transport properties established PtSn_4_ as a distinctive platform by exhibiting strong regulation of the two-carrier transport behavior under both a thermal gradient and a magnetic field. PtSn_4_ is a highly conductive topological semimetal with low electrical resistivity at liquid helium temperatures, which is comparable to that of many well-established metals or topological semimetals [44]. Hall effect measurement of PtSn_4_ revealed a two-carrier behavior with both high electron and high hole mobilities. At zero magnetic field, the thermopower is close to zero, and the thermal conductivity is orders of magnitude higher than that of good thermoelectric semiconductors. These features indicate that PtSn_4_ has a poor thermoelectric performance.

However, all these transport properties change significantly when a magnetic field is applied. Large Seebeck and Nernst power factors and a huge suppression of the thermal conductivity are simultaneously realized under various magnetic fields, providing a good example for decoupling the electrical and thermal transport properties of semimetals. These dramatic changes in the transport properties are the result of the regulation of the transport behavior of the two types of carriers under a magnetic field. At zero magnetic field, the thermal gradient drives the accumulation of both electrons and holes on the cold side, which balance each other's contributions to the Seebeck thermopower. When a magnetic field is applied, the electrons and holes exhibit different transport behaviors, which makes it possible to realize strong thermoelectric responses in both the longitudinal and transverse directions. These results on PtSn_4_ provide a paradigm for tuning highly conductive semimetals for both longitudinal and transverse thermoelectric conversion by applying a magnetic field. Interestingly, some recent work had paid attention to the relationship between thermoelectric performance and internal magnetic interactions, for example, superparamagnetic behavior [45], paramagnon drag [46], and spin fluctuation [47]. These works together with our current study on PtSn_4_ demonstrate that introducing magnetic interaction as an additional influence on the transport properties, either by applying an external magnetic field or by introducing an internal magnetic moment, could open a new direction in thermoelectric research.

## 4. Experimental Methods

Single crystals of PtSn_4_ were grown from a Sn-rich binary melt as described in the literature [31]. The high-purity starting elements, Pt (shot, 99.99%) and Sn (shot, 99.999%), were mixed with an initial stoichiometry of Pt_4_Sn_96_; the mixture was placed in an alumina crucible, which was sealed in a quartz tube under partial Ar pressure. The quartz tube was heated to 600°C over a period of 5 h and held at that temperature for 20 h. Next, it was slowly cooled to 350°C over a period of 60 h. The excess Sn flux was removed using a centrifuge at 400°C. After centrifugation, the remaining flux on the surface was removed by mechanical polishing.

Powder X-ray diffraction measurement of PtSn_4_ was performed using Cu K*α* radiation at room temperature to identify the phase purity and crystal structure. An image-plate Huber G670 Guinier camera was used in a diffraction range of 10° ≤ 2*θ* ≤ 100° in steps of 0.005°. Scanning electron microscopy (SEM) with energy-dispersive X-ray spectrometry (EDXS) was used for elemental analysis. Single-crystal X-ray diffraction measurements were performed using a Bruker D8 Venture diffractometer with Mo K*α* radiation and the SHELX97 software was used for structure refinement [48]. More information about the crystallographic and refinement parameters can be found in our previous work [49], where the studied PtSn_4_ single crystal is from the same batch with the current work. The crystal structure is described by the centrosymmetric space group *Ccca*, in agreement with the literature [50]. Figure 1(a) shows the layered crystal structure of PtSn_4_, which consists of PtSn_4_ slabs constructed from eight-fold Sn-coordinated Pt atoms. The layered structure is visible in the SEM results, as shown in Figure S2. The layered nature of PtSn_4_ allows for slight disorder in the ac plane, i.e., a small misalignment between consecutive PtSn_4_ layers.

The powder X-ray diffraction pattern of PtSn_4_ is shown in Figure S3. The diffraction peaks can be well indexed to orthorhombic structure, in good agreement with the single-crystal X-ray diffraction results [49]. No other obvious phases are observed. The actual composition of the PtSn_4_ single crystal is determined by EDXS at seven randomly selected positions; the result agrees with the nominal composition within the instrument error, as shown in Table S2. High-resolution transmission electron microscopy (HRTEM) (Figure S4) of a 6.7 *μ*m × 4.4 *μ*m lamella showed the good crystallinity of the PtSn_4_ sample (Figure S5).

The temperature-dependent thermal conductivity and thermopower under a magnetic field were jointly measured adiabatically by the one-heater, two-thermometer configuration using the thermal transport option of the PPMS (Quantum Design), in which the sample was placed so that the magnetic field was perpendicular to the heat flow. The thermometers were calibrated under the magnetic field using the PPMS MR calibration wizard before the thermal transport measurements. To ensure uniform heat flow through the bar-shaped sample, two gold-plated copper leads were attached to both ends of the sample using silver epoxy and then connected to the heater and sink, respectively. Two other copper leads were wrapped around and glued to the sample with silver epoxy for detecting Δ*V* and Δ*T*. The applied temperature gradients were approximately 1%–3% of the base temperature. We then investigated the possible effect of the thermal contact resistances and demonstrated the need for careful contact preparation. After careful contact preparation, as described above, we investigated samples with lengths ranging from 4.5 to 7 mm, but similar cross-sections and contact areas. We obtained consistent results for all the samples at any magnetic field or temperature investigated, and therefore did not observe any indication of diminished thermal conductivity owing to contact. The Nernst thermopower was also measured adiabatically in the one-heater, two-thermometer configuration using the PPMS. The Nernst signal was estimated as, *S*_*xy*_ = *L*_*x*_*V*_*y*_/*L*_*y*_Δ*T*_*x*_, where *L*_*x*_ is the distance between the two temperature leads, *L_y_* is the distance between the two voltage wires, *V*_*y*_ is the transverse electric voltage, and Δ*T*_*x*_ is the measured temperature difference. The measured raw Nernst thermopower data were antisymmetrized to correct for contact misalignment.

Both the longitudinal and Hall resistivities were measured by a standard four-probe method using the AC transport option in the PPMS system with an applied AC current of 16 mA. Point contacts for Hall voltage probes were obtained by spot-welding 25 *μ*m platinum wires. For the current and longitudinal voltage probes, linear contacts were made using silver paint and platinum wires. To correct for contact misalignment, the measured Hall resistivity was field antisymmetrized. For all the transport measurements, the magnetic field was applied along the *b* axis, which was perpendicular to the *a*–*c* plane.

## Supplementary Materials

Supplementary Materials Fermi surface, SEM images, TEM images, XRD pattern, and EDX results.
