# Supplementary material for: Largely Suppressed Magneto-Thermal Conductivity and Enhanced Magneto-Thermoelectric Properties in PtSn4
Source: Research (Wash D C). 2020 Apr 7;2020:4643507. doi: 10.34133/2020/4643507 (PMC7166253; doi:10.34133/2020/4643507)
Supplement: Supplementary Materials — Fermi surface, SEM images, TEM images, XRD pattern, and EDX results. [file 4643507.f1.docx]

**Supporting Information**

**Largely Suppressed Magneto-Thermal Conductivity and Enhanced Magneto-Thermoelectric Properties in PtSn_4_**

*Chenguang Fu^1*^, Satya N. Guin^1^, Thomas Scaffidi^2^, Yan Sun^1^, Rana Saha^3^, Sarah J. Watzman^4^, Abhay K. Srivastava^3,5^, Guowei Li^1^, Walter Schnelle^1^, Stuart S.P. Parkin^3^, Claudia Felser^1^, Johannes Gooth^1*^*

^1^Max Planck Institute for Chemical Physics of Solids, 01187 Dresden, Germany.

^2^Department of Physics, University of California, Berkeley, CA 94720, USA.

^3^Max Planck Institute of Microstructure Physics, 06120 Halle, Germany.

^4^Department of Mechanical and Materials Engineering, University of Cincinnati, Cincinnati, OH 45219, USA.

^5^Institute of Physics, Martin Luther University Halle-Wittenberg, Halle (Saale) D-06120, Germany.

∗ Corresponding Author

chenguang.fu@cpfs.mpg.de; johannes.gooth@cpfs.mpg.de


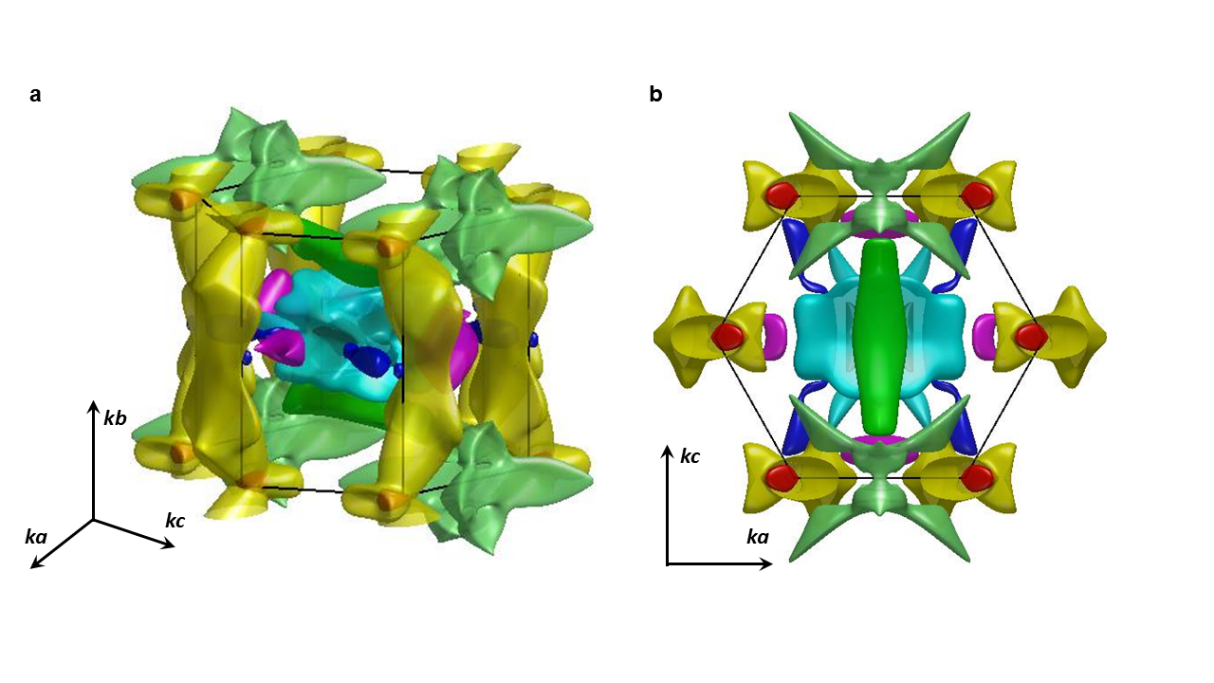

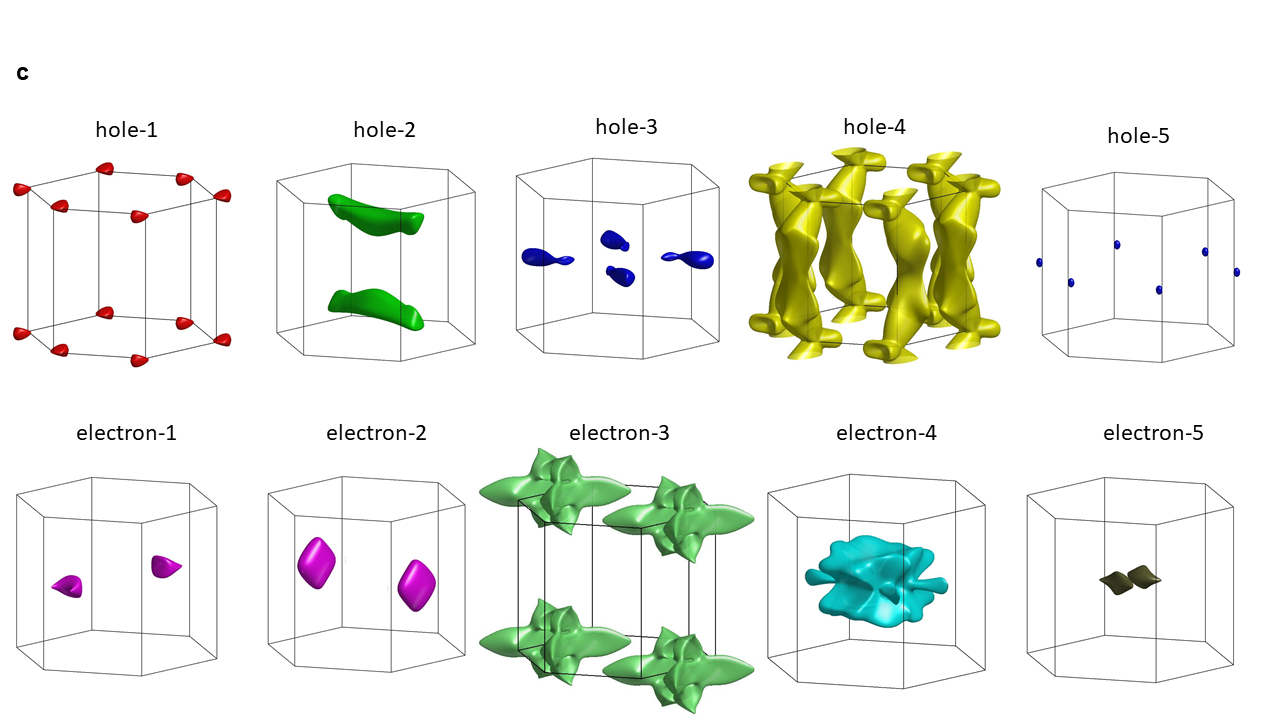


Figure S1. The Fermi surface of PtSn_4_ is calculated using Density Functional Theory. a, Side view of the whole Fermi surface. b, Top view of the whole Fermi surface. c, Individual hole (upper row) and electron (lower row) pockets.

Table S1. The fitting parameters used in Figure 3a.

| *T* (K) | *κ*_ph_(*T*) (Wm^-1^K^-1^) | *κ*_e_(*T*) (Wm^-1^K^-1^) | *s* | *η*^1/^*^s^* (T^-1^) |
| --- | --- | --- | --- | --- |
| 2 | 0.272 | 166.4 | 1.588 | 6.340 |
| 6 | 1.533 | 313.8 | 1.438 | 4.953 |
| 10 | 5.363 | 241.9 | 1.317 | 3.157 |
| 14 | 6.884 | 152.5 | 1.082 | 1.842 |
| 20 | 10.51 | 80.74 | 1.004 | 0.716 |
| 30 | 18.13 | 37.30 | 1.163 | 0.278 |


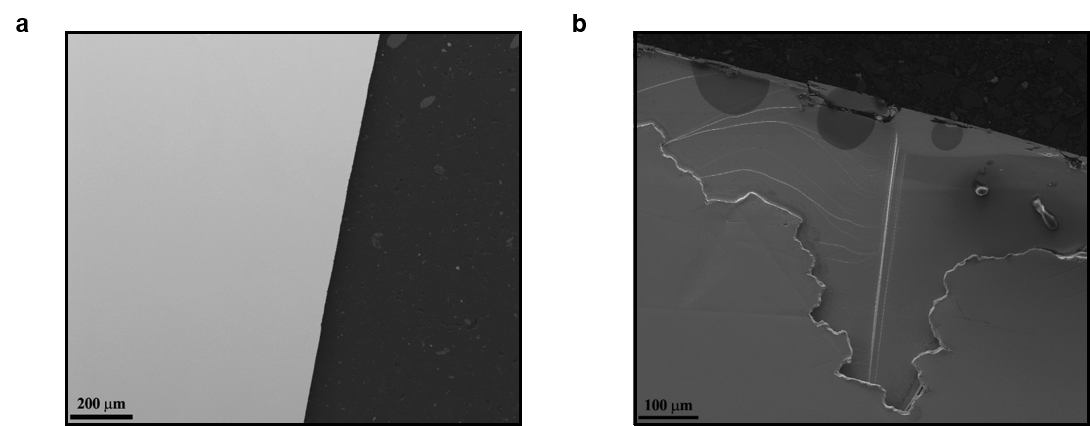


Figure S2. a. SEM back scattering and b. secondary electron images for the PtSn_4_ single crystal. The layered structure is clearly observed.


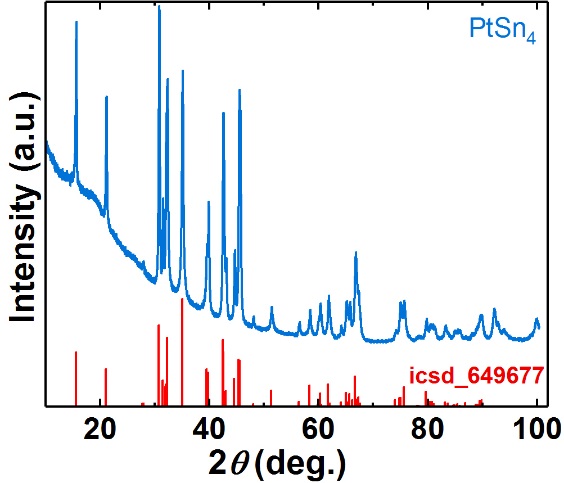


Figure S3. Power X-ray diffraction pattern of PtSn_4_.


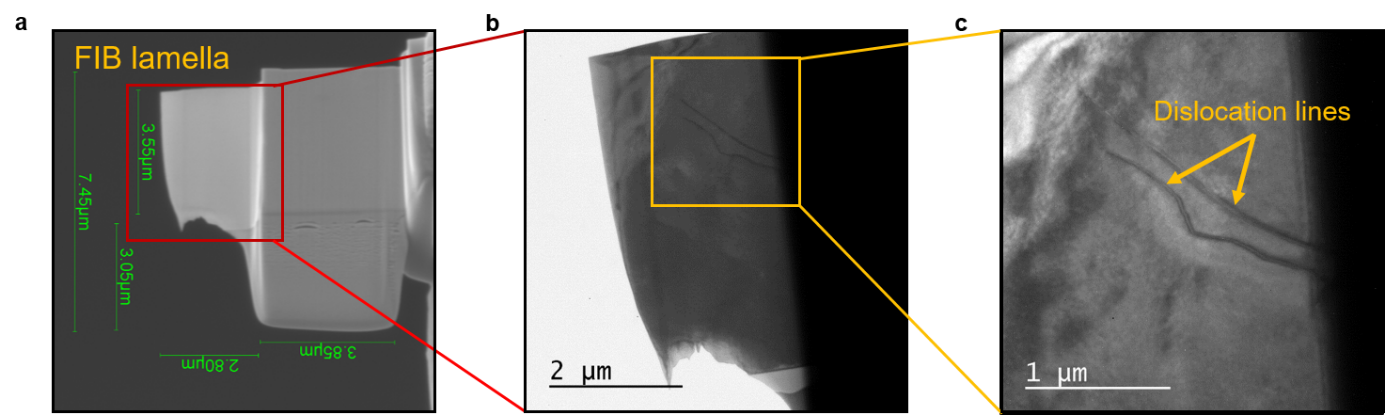


Figure S4. a. Scanning electron microscopy image showing the sample prepared by Focussed Ion Beam technique (FIB). The dimensions of the thin slice are inserted. b. Corresponding TEM micrograph of the thin area. “Diffraction contrast technique” was applied, which allows the identification of crystal defects. c. The magnified area shows to parallel dark lines, which were identified as dislocation.

Figure S5. a. Electron diffraction pattern received from the thin sample area. It correlates to a [010] crystal orientation. The corresponding reflections are indicated. b. TEM overview image. c. High-resolution TEM image showing the crystal lattice structure by (002) lattice planes with a distance of 0.32 nanometer.

Table S2. Atomic percentage of PtSn_4_ single crystal at seven randomly selected positions detected by EDXS.

|  | **Position 1** | **Position 2** | **Position 3** | **Position 4** | **Position 5** | **Position 6** | **Position 7** | **Average** |
| --- | --- | --- | --- | --- | --- | --- | --- | --- |
| Pt | 22.11% | 21.58% | 21.89% | 21.00% | 22.18% | 20.87% | 21.16% | 21.54% |
| Sn | 77.89% | 78.42% | 78.11% | 79.00% | 77.82% | 79.13% | 78.84% | 78.46% |
